# Effectiveness of nutrition education in enhancing knowledge and attitude of pupils on choice of school mid‐day meal in Ibadan, Nigeria

**DOI:** 10.1002/fsn3.3359

**Published:** 2023-04-17

**Authors:** Akindele Abimibayo Adeoya, Adetoun Tayewo Akinwusi, Ryoichi Nagatomi

**Affiliations:** ^1^ Medicine and Science in Sports and Exercise Laboratory Graduate School of Medicine Tohoku University Sendai Japan; ^2^ Division for Interdisciplinary Advanced Research and Education Advanced Graduate School Tohoku University Sendai Japan; ^3^ Department of Human Kinetics and Health Education, Faculty of Education University of Ibadan Ibadan Nigeria; ^4^ Division of Biomedical Engineering for Health and Welfare Graduate School of Biomedical Engineering Tohoku University Sendai Japan

**Keywords:** dietary pattern, food choices, mid‐day meals, nutrition education, pupils

## Abstract

Children's food choices affect their nutritional health, development, and well‐being. In Nigeria, school lunch is generally unregulated; the food menu is usually at the discretion of individual vendors forcing pupils to make unsupervised food choices. This study examined the effectiveness of 6‐week nutrition education in enhancing pupils' knowledge and attitude on the school mid‐day meal choices in Ibadan, Nigeria. A pre‐test/post‐test, quasi‐experimental study was conducted with 100 pupils in 4th and 5th grades in public primary schools. Multistage sampling was used to select the participants. A nutrition education module and a questionnaire were used to collect the data. Frequency counts and *t*‐test were used for statistical analysis. Findings revealed the following dietary pattern: 44 of the pupils preferred junk food (mean 41.5, SD = 12.9), 37 were inclined toward protein‐rich food (mean 37.7, SD = 12.5), 32 favored food items dense in carbohydrates (mean 34.4, SD = 9.5), and 11 showed a preference toward food with high vitamins and minerals (mean 28.4, SD = 7.5). Nutrition knowledge (*t* = 6.4, df = 99, *p* < .05); (pre‐test: $\bar{X}$ = 20.9 and SD = 1.0; post‐test: $\bar{X}$ = 22.8, SD = 2.8) and attitude toward choice of meal showed statistically significant differences (*t* = 4.9, df = 99, *p* < .05; pre‐test: $\bar{X}$ = 13.5 and SD = 6.8; post‐test: $\bar{X}$ = 18.2, SD = 7.2). We recommend that the Ministry of Education should prepare a standard lunch menu for all schools and the school authorities should enforce it through the schools' food vendors.

## Graphical abstract

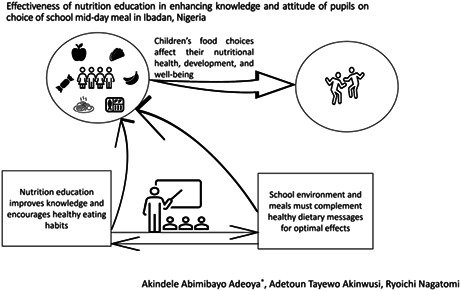

## 1 INTRODUCTION

Nutrition is pivotal to our life, health, and development. Early establishment of healthy eating behavior helps prevent or reduce the likelihood of diseases and long‐term health issues (Ergin et al., 2007; Nicklas et al., 2008; Weichselbaum & Buttriss, 2014). The promotion and provision of a healthy diet during childhood not only contributes to better mental, social, physical, and dental health in early years but also lays the foundation for improved health throughout life, thereby leading to a longer life expectancy and better quality of life (Abimibayo Adeoya et al., 2022; Ayele et al., 2013; Weisenberger & Klemm, 2022). In addition, it is the ideal period to shape and reinforce healthy eating and lifestyle behaviors to avoid the risk of nutrition‐related problems in adulthood.

Diet quality is an important link between food security and nutrition. The number of people worldwide who could not afford to eat healthy food increased to approximately 3.1 billion in 2022, of which a wholesome diet was out of reach for 27 million additional individuals in sub‐Saharan Africa (FAO, IFAD, UNICEF, WFP, & WHO, 2022). Children in low‐ and lower‐middle‐income countries and poorer households bear the burden of stunting, wasting, and anemia (Abimibayo Adeoya et al., 2022; UNICEF, 2019). Poor nutrition impedes the well‐being of the masses and undermines the prosperity of a nation since malnutrition impairs educational achievement and economic productivity. It costs governments and families enormous sums of money to treat related diseases (Government of Uganda, 2011; UNOCHA, 2014). Globally, Nigeria has the second highest acute malnutrition rate with an estimated 3.78 million children suffering from wasting. The Nigerian National Nutrition and Health Survey 2018 revealed that global acute malnutrition, moderate acute malnutrition, and severe acute malnutrition prevalence for Nigerian children under the age of 5 years were 7.0%, 5.5%, and 1.5%, respectively (National Bureau of Statistics, 2018). The increasing global rate of malnutrition and diet‐related chronic diseases that are of grave concern necessitated the UN Food Systems Summit 2022 to reiterate the urgent need for an effective food system and diet transformation to promote better and equitable access to nutrition (Development Initiatives, 2022).

Influenced by social, environmental, and biological factors, food preferences remain dynamic throughout an individual's life span (Ventura & Worobey, 2013). Modern diet that is particularly nutritionally poor consists of meals consumed away from home, such as fast food, instant food, energy‐dense snacks, and soft drinks. As they are generally higher in calories, saturated fat, salt, and cholesterol and lower in fiber, calcium, and iron, they can lead to severe malnutrition (Guthrie et al., 2002; Maslin et al., 2015; Nordström & Thunström, 2015; Scaglioni et al., 2018). This kind of diet predisposes people to chronic diseases that can cause great suffering, health problems, and premature death (British Medical Association, 2015). Unfortunately, these junk food items are often more appealing and accessible to children. Therefore, it is important for children to know the benefits of good nutrition and develop healthy eating habits at an early age. A controlling approach, including restrictions, pressure to eat healthy food, and use of rewards, has been reported to have a negative impact on children's dietary behavior (DeCosta et al., 2017; Galloway et al., 2005; Gregory et al., 2011; Sleddens et al., 2010). In contrast, they effectively respond to dietary messages through an empowerment approach, that is, nutrition education and motivation (Contento et al., 1995; Glorioso et al., 2020; Obidoa et al., 2021; Wall et al., 2012; Wolfenden et al., 2017). Moreover, Buttriss (2002) pointed out that empowering a child with the knowledge, understanding, and skills to make appropriate food choices and develop a positive attitude toward food and diet‐related issues is critical. Schools are effective and engaging environments for nutrition education to improve children's health and nutrition to reduce the risk of future chronic diseases (McKenna, 2000; Medeiros et al., 2022; Weichselbaum & Buttriss, 2014). This is owing to the fact that schools have the potential to effectively target a large number of children and parents simultaneously over an extended period of time in a relatively low‐cost manner. Eating habits develop at an early age. Consequently, for children to develop the knowledge, understanding, and skills necessary to make appropriate food choices and develop a positive outlook toward food and health, messages about a healthy lifestyle must be communicated in a clear and consistent manner (Weichselbaum & Buttriss, 2014). Particularly, White (2021) opined that primary schools provide an ideal setting for more effective education about healthy eating. This can lead to the pupils' ability to make informed, positive food choices and manage their diet.

In Nigeria, school feeding service is a critical component of the school health program and is part of the national school health policy aimed to provide pupils with a daily supplementary adequate meal. This system aims to not only improve their health but also their nutritional status for the achievement of effective and wholesome learning (Federal Ministry of Education Nigeria, 2006). It is to ensure that a considerable percentage of pupils who do not receive proper nutrition at home can at least get enough food at school to promote health and learning (Moronkola, 2012). Malnutrition constituted a major health issue for school children in Nigeria (Oninla et al., 2007). A situational analysis conducted by the World Bank Group identified health and nutrition problems among school‐aged children in addition to gaps in the existing school nutrition and health services in Nigerian schools (World Bank Group, 2015). Several studies have also reported the double burden of malnutrition (DBM) among Nigerian school‐aged children (Alamu et al., 2020; Ene‐Obong et al., 2012; Umeokonkwo et al., 2020; Wariri et al., 2020). For instance, a cross‐sectional survey in two Nigerian states revealed the prevalence rate of 34.9%, 13.5%, 10.3%, and 11.4% for stunting, underweight, thinness, and overweight, respectively, among school‐aged children and adolescents as well as DBM at both individual and population levels (Adeomi et al., 2021).

Decades of neglect occasioned by a deficit of adequate human and material resources, poor motivation and remuneration for teachers, lack of accountability, monitoring, and poor budgetary allocation, and incessant strike actions have undoubtedly affected the quality of education in Nigeria. Therefore, pupils enrolled in public schools, specifically primary schools, in Nigeria are mainly children from low socio‐economic households who could not afford a paid alternative. Often, their parents have little or no education, thereby limiting the possibility of awareness about nutrition as well as the financial capacity to outsource quality and healthy diets for themselves and their children. Ugochukwu et al. (2014) confirmed that lunch packs of public primary school pupils contain poor‐quality food. In addition, in Nigeria, school lunch is mostly unregulated, and the food menu is typically decided by individual vendors, thus compelling pupils to make their own food choices. In some public primary schools in Ibadan, it has been observed that for the mid‐day meal, pupils prefer junk food, such as chin chin (fried snack), meat pie, buns, macaroni, ice lolly, and biscuits to local quality food, such as beans, rice, yam porridge, vegetables, and fruits. Olanipekun et al. (2012) noted the prevalence of malnutrition among the average school‐age children in Ibadan. A quick search of publications using PubMed and Scopus with the following search string “nutrition education intervention in Nigeria schools” “school‐based nutrition education in Nigeria” showed seven school‐based studies on nutrition education intervention in Nigeria (Anetor et al., 2012, 2013; Desmennu & Arulogun, 2019; Eboh & Boye, 2006; Ezezika et al., 2018; Ogunsile, 2021; Ogunsile & Ogundele, 2016), whereas a reference from a relevant search manually yielded an additional article (Kukoyi & Amosu, 2020). Most of these studies targeted secondary school pupils while only one (Eboh & Boye, 2006) considered primary school children in the Niger Delta region despite their vulnerability. It is, therefore, essential to empower this population with adequate awareness of affordable and local quality food, motivation, and skills to develop an appropriate attitude toward nutrition and make suitable food choices for a healthy and active life. Thus, this study aimed to investigate the effectiveness of nutrition education in enhancing the knowledge and attitude of pupils on the choice of school mid‐day meals in Ibadan, Nigeria.

### 1.1 Key message

Nutrition education improves knowledge and encourages healthy eating habits. School environment and meals must complement healthy dietary messages for optimal effects.

### 1.2 Research purpose

This study aimed to examine the effectiveness of nutrition education in enhancing the knowledge and attitude of pupils on the choice of school mid‐day meals in Ibadan, Nigeria, from the perspective of health education and promotion. Specifically, it investigated the following aspects:

1. Dietary pattern of primary school pupils in Ibadan.
2. Their knowledge of the relationship between food choices and health.
3. The effects of nutrition education on the knowledge and attitude of pupils toward school mid‐day meal choices in Ibadan.

## 2 MATERIALS AND METHODS

### 2.1 Study design and participants

This study adopted the pre‐test/post‐test quasi‐experimental research design. One hundred pupils in grades 4 and 5 in public primary schools in the Ibadan metropolitan area in Nigeria were recruited using multistage sampling. Fishbowl sampling technique with replacement was used to select three of the five Local Government Areas (LGA) in Ibadan. Proportionate sampling method was used to choose 3% of the total number of public primary schools in each of the three LGAs selected: two schools each in North and Southeast Ibadan and one school in the Northwest region. Purposive sampling was used to choose pupils in primary 4th and 5th grades in each of the selected schools. A total of 20 pupils who volunteered were selected from each school. Based on gender, an equal allocation was done in the sample to include five boys and five girls in every class from each of the schools selected.

### 2.2 Instruments and procedure

The instruments, namely a nutrition education module and questionnaire, were presented for content validation to experts in the Department of Human Kinetics and Health Education, Faculty of Education as well as the Department of Human Nutrition, Faculty of Public Health, and College of Medicine both in the University of Ibadan. Their comments and suggestions were carefully incorporated to improve the quality of the instruments. Measurement properties of the instruments were confirmed through a pilot study of the school children in Ibadan who were not included in the study. Using test–retest method, the reliability of the questionnaire and nutrition education module were verified to be adequate, realistic, understandable, culturally appropriate, visually appealing, and motivating. Participants were given pre‐test questionnaires followed by a 6‐week (30‐min lesson twice a week) training on nutrition education. According to previous research, 30 min of nutrition education per week is effective in exacting behavioral change and improving the nutrition knowledge of primary school pupils (DeVault et al., 2009; Friel et al., 1999; Guenther et al., 2018). Post‐test questionnaires were given immediately after the intervention, and they were promptly collected to prevent interference effect.

### 2.3 Nutrition education module

The nutrition education module is a resource material with appropriate information on nutrition education for school‐age pupils. It comprises a comprehensive lesson plan containing step‐by‐step teaching procedures, such as instructional objectives and methods, key message, teaching aids, target population, duration, and evaluation for effective teaching and learning.

### 2.4 Questionnaire

The questionnaire was divided into two sections: Sections A and B. Section A comprised the demographic characteristics of the participants, including gender (male and female), grade (primary 4 and 5), age (6–8, 9–11, 12–14, and 15 years and above), and socio‐economic status, that were estimated according to the amount given to the pupils by their parents for their personal expenses in school (none, $0.011–$0.055 (₦5–₦25), $0.066–$0.11 (₦30–₦50), $0.12–$0.15 (₦55–₦70), $0.17 (₦75), and above). Section B consisted of the food items commonly sold in schools that were classified into four groups based on their main nutritional benefits: proteins (beans, egg, meat, and fish), carbohydrates (potatoes, plantain, rice, and yam porridge), vitamins and minerals (fruits and vegetables, e.g., cherries, mangoes, and oranges), and junk food items (buns, puff puff/fried dough, biscuits, ice lolly, macaroni/pasta, spaghetti, popcorn, and minced pie). Participants were asked to indicate their preferred school mid‐day meals to determine their attitude and identify the nutritional benefits of each food for knowledge.

### 2.5 Data analysis

Data collected were entered into Excel software and analyzed using Statistical Package for Social Sciences (SPSS version 20). The data generated were processed and analyzed using frequencies and paired sample *t*‐test to assess the overall effect of the intervention at 0.05 alpha level.

### 2.6 Ethical consideration

Approval was obtained and a letter of introduction was collected from the Head of the Department of Human Kinetics and Health Education, University of Ibadan, to be handed over to the authorities of the selected primary schools in Ibadan for permission to carry out the study. Subsequently, the participants were selected, and the purpose of the study was duly explained to them. They were given consent forms that had to be signed by their parents. Only those pupils who returned the duly signed form were permitted to participate in the research.

## 3 RESULTS

The demographic characteristics of the participants are shown in Table 1.

**TABLE 1:** Demographic characteristics of the participants.

Variable	*n*
Grade	
Primary 4	50
Primary 5	50
Gender	
Male	50
Female	50
Age (years)	
6–8	8
9–11	66
12–14	24
15 and above	2
Pocket money (for school expenses) in US dollars ($) and Nigerian Naira (₦) equivalent	
None	26
$0.011–$0.055 (₦5–₦25)	10
$0.066–$0.11 (₦30–₦50)	38
$0.12–$0.15 (₦55–₦70)	22
$0.17 (₦75) and above	4

*Note*: *n* = sample size per category.

Based on gender and grade, the pupils were equally distributed in the sample. Most pupils (66) were in the age group of 9–11 years. Thirty‐three pupils (the majority) received a daily allowance of 0.066–0.11 US dollars (30–50 naira) from their parents for school expenses, while 22 pupils got none.

Table 2 shows the various dietary patterns of public primary school pupils in Ibadan. The results indicate that among participants 44 preferred junk food (mean = 41.5, SD = 12.9) while 37 were inclined toward protein‐rich food (mean = 37.7, SD = 12.5). Approximately 32 of the participants favored food that was dense in carbohydrates (mean = 34.4, SD = 9.5), whereas 11 of the participants leaned toward food with high vitamins and minerals (mean = 28.4, SD = 7.5).

**TABLE 2:** Dietary pattern of public primary school pupils in Ibadan, Nigeria.

Dietary pattern	Preference (*n*)		Mean	SD	Ranking
	No	Yes			
Junk food items	56	44	41.5	12.9	1st
Proteins	63	37	37.7	12.5	2nd
Carbohydrates	68	32	34.4	9.5	3rd
Vitamins and minerals	89	11	28.4	7.5	4th

We can see, for instance, that 92 of the participants preferred rice, of which 80 were aware that it is a source of carbohydrates, while 20 pupils lacked this knowledge. Their choices and knowledge regarding the nutritional value of the food items are presented in detail in Table 3. This result implies that pupils in primary schools in Ibadan did not adequately understand the relationship between choice of mid‐day meal and health, especially sources of proteins and vitamins and minerals.

**TABLE 3:** Pre‐test knowledge on the relationship between choice of school lunch and health.

Food	Pupils' dietary choices (*n*)		Level of knowledge about the nutritional value of meals			
	Yes	No	Carbohydrates	Proteins	Vitamins & minerals	Fat & oil
Rice	92	8	80	8	4	8
Beans	90	10	20	74	3	3
Porridge	96	4	58	14	8	20
Spaghetti	96	4	84	4	4	16
Sweet potatoes	96	4	62	30	4	4
Plantain	100	0	72	20	4	4
Puff puff/fried dough	94	6	78	10	12	0
Buns	96	4	76	4	10	10
Minced pie	96	4	40	31	20	9
Ice lolly	88	12	76	14	5	5
Macaroni	100	0	81	10	5	4
Biscuits	100	0	80	10	5	5
Popcorn	94	6	78	10	12	0
Oranges	88	12	20	41	39	0
Mangoes	94	6	10	41	39	10
Cherries	96	4	10	29	20	41
Vegetables	96	0	10	29	41	20
Eggs	100	0	10	48	30	12
Meat	98	2	8	52	10	30
Fish	100	0	18	52	20	10

The paired *t*‐test and pre‐ and post‐test scores of the participants on nutrition knowledge and attitude are presented in Table 4. Findings revealed that there were significant effects of nutrition education on the knowledge and attitude of the participants toward the choice of mid‐day meals in school.

**TABLE 4:** Effects of nutrition education on knowledge and attitude of pupils toward school mid‐day meals.

Nutrition	*n*	Mean	SD	*t*‐cal.	*t*‐crit.	df	*p*
Knowledge							
Pre‐test		20.9	1.0	6.4			
Post‐test	100	22.8	2.8		1.99	99	<.05
Attitude							
Pre‐test		13.5	6.8	4.9			
Post‐test		18.2	7.2				

The result of the *t*‐test analysis on the gender effect of the pupils is presented in Table 5. Results indicate a significant gender effect on the knowledge and attitude toward choice of school mid‐day meals. While males scored higher than females on knowledge, females had higher scores on the attitude component than males.

**TABLE 5:** Gender effect on nutrition knowledge and attitude of pupils.

Gender effect	*n*	Mean	SD	*t*	df	*p*
Knowledge						
Male	50	21.3	1.3	3.9		
Female	50	20.5	0.5		98	<.05
Attitude						
Male	50	16.7	6.6	2.1		
Female	50	19.6	7.5			

## 4 DISCUSSION

This study aimed to explore the dietary pattern of pupils, their knowledge of the correlation between their choice of mid‐day meal and health, and the effects of nutrition education intervention on their knowledge and attitude toward the choice of school mid‐day meals. Findings suggest that most pupils' choice of school mid‐day meals consisted of junk food items, thus confirming previous findings that children have a natural inclination toward sweet taste and unhealthy snacks (Medeiros et al., 2022; Noble et al., 2003; Olumakaiye et al., 2010). This may also be associated with affordability. Children's preference for junk food could primarily be the consequence of the prevailing socio‐economic factors, which are a major determinant of the nutritional status in Nigeria (Akerele et al., 2014; National Population Commission (NPC) (Nigeria) & ICF international, 2014; Ndukwu et al., 2013; Okafor et al., 2021). Children attending public primary schools in Nigeria typically belong to a low socio‐economic background, and most receive negligible or zero food allowance from their parents/guardians. The largely independent school vendors, in a bid to obtain profits and simultaneously make the food affordable, tend to sell food items that are low in both quality and quantity, especially junk food. For instance, the buns sold only contain flour, yeast, and a low amount of sugar or artificial sweeteners without any milk or eggs, and the oil for frying food is repeatedly used for an extended period. This is evident from the texture, taste, and appearance of the food. Furthermore, the allowance received by most pupils allowed them to only buy snacks (biscuits and buns) even if they preferred a better option. It is particularly worrisome that despite the long hours spent in school, a substantial number of children attend school without any food allowance or provision for meals by the parents, schools, or the government. This could lead to adverse consequences, including malnutrition, absenteeism, early school dropout, poor academic performance, and low cognitive development.

Pupils in Ibadan did not understand the link between the choice of school mid‐day meals and health and were unable to identify the sources of proteins as well as vitamins and minerals. This is in line with the findings of a previous study by Ogwumike and Ozughalu (2018) on child poverty and deprivation in education, nutrition, health, water, and sanitation, which are rather evident in Nigeria. Similar to most European countries (Weichselbaum et al., 2011), nutrition is integrated into subjects, such as health and physical education, home economics, and agricultural science in Nigerian primary and junior secondary schools. In addition, Adu et al. (2015) noted that the time spent on teaching nutrition in these integrated subjects is lower than recommended. In a review of the Nigerian school health program, which includes school health services; healthful school environment; school feeding services; skill‐based health education; and school, home, and community relationships, Dania and Adebayo (2019) found that the Nigerian school health program has largely remained at the policy level. Incessant strike actions and insufficient human and material resources in public schools may have contributed to inadequate nutrition knowledge.

The study results indicate the positive effects of nutrition education on pupils' knowledge and attitude toward choice of mid‐day meals. This conforms to the results of several prior studies (Anetor et al., 2013; Desmennu & Arulogun, 2019; Eboh & Boye, 2006; Jadgal et al., 2020; Kukoyi & Amosu, 2020; McCullough et al., 2004; Medeiros et al., 2022; Ogunsile, 2021; Wall et al., 2012; Wolfenden et al., 2017). Moreover, it confirms the previous perspective that 30 min of nutritional education per week in primary schools can have numerous positive effects on pupils' health and prosocial behavior (Guenther et al., 2018). However, the sustainability and long‐term influence of the intervention on attitude are concerning because the meals offered at some schools did not complement the healthy eating messages. The largely unregulated nature of the meals sold by school vendors and unrestricted access of pupils to unhealthy snacks within and outside the school environment, especially after school hours, could limit the long‐term impact of the intervention. This is due to the fact that children's dietary habits are essentially dependent on what is easily available and accessible to them (DeCosta et al., 2017). In addition, effective learning entails children having the opportunity to apply what was learned in the classroom and the consistency of messages across the entire school environment (Lakin & Littledyke, 2008). Therefore, access to healthy food choices within the school environment may boost efforts to improve nutrition in school‐aged children.

## 5 RELEVANCE AND CONTRIBUTION OF THE STUDY

The study has theoretical relevance as it provides insights into the current situation of mid‐day meals in Nigerian schools and the dietary preferences of children in this country. It has practical relevance as it offers valuable suggestions to promote children's awareness of nutrition and enhance their understanding of the correlation between diet and health.

## 6 CONCLUSION AND RECOMMENDATIONS

Nutrition education is effective in enhancing children's knowledge and attitude toward nutrition and healthy diet. However, the meals offered at some schools did not complement the healthy eating messages. It is recommended that the Ministry of Education in collaboration with the Ministry of Health prepares a standard lunch menu for all schools and the school authorities implement it through school food vendors. School gardens and other commonly frequented areas must be utilized for the strategic placement of nutrition messages that should be promoted in schools.

### 6.1 Limitations

This research has certain limitations. Some commonly sold food items were excluded from this research as they were not available during the study period due to seasonal variations. Furthermore, although efforts were made to read and explain each item on the questionnaire because a few participants could not read, it may have affected their responses and, therefore, the study outcome.

## AUTHOR CONTRIBUTIONS

**Akindele Abimibayo Adeoya:** Conceptualization (lead); data curation (lead); formal analysis (equal); funding acquisition (lead); investigation (lead); methodology (lead); project administration (equal); resources (lead); supervision (equal); validation (equal); visualization (lead); writing – original draft (lead); writing – review and editing (equal). **Adetoun Tayewo Akinwusi:** Conceptualization (equal); formal analysis (equal); investigation (supporting); methodology (equal); project administration (equal); resources (equal); supervision (lead); validation (equal); writing – original draft (supporting). **Ryoichi Nagatomi:** Funding acquisition (equal); visualization (equal); writing – review and editing (lead).

## ACKNOWLEDGEMENTS

We express our appreciation to the headmaster/headmistress, staff, students, and parents of the participants in the selected schools in Ibadan for their support and understanding throughout the intervention program.

## FUNDING INFORMATION

This work was supported by JST SPRING, Grant Number JPMJSP2114.

## CONFLICT OF INTEREST STATEMENT

The authors declare no conflicts of interest.

## Data Availability

The data that support the findings of this study are available from the corresponding author upon reasonable request.
